# Supplementary material for: Content-rich biological network constructed by mining PubMed abstracts
Source: BMC Bioinformatics. 2004 Oct 8;5:147. doi: 10.1186/1471-2105-5-147 (PMC528731; doi:10.1186/1471-2105-5-147)
Supplement: Additional File 5 — The original Chilibot query results of the term "long-term potentiation (LTP)" and 22 other terms, limiting the latest references analyzed to the years 1990, 1995, 2000, and 2004. [file 1471-2105-5-147-S5.bz2 › chilibotAdditionalFile5/ltp1995/html/TAU_SYNAPTOPHYSIN.html]

 


 **TAU** and **SYNAPTOPHYSIN** 
  
Found 25 abstracts in PubMed,  **25 abstracts were retrieved and analyzed**.  


---

 Search Google  |
 PDF files only 
|  EDU domain only 

---

**Interactive relationship** (e.g. stimulation, inhibition, etc)

- Three dimensional 3 D reconstructions of serial optical sections showed that the  **tau**  positive tortuous axons clustered in the neuritic plaques were often continuous with  **synaptophysin**  positive distended terminals.  Ref: 8229081 J Neuropathol Exp Neurol, 1993
- The olfactory and peripheral neurons of the olfactory mucosa in both AD and control patients typically exhibited immunoreactivity to neurofilament NF triplet proteins, including both phosphorylated and non phosphorylated epitopes, as well as to  **synaptophysin** , but lacked reactivity to other intermediate filament proteins, microtubule associated protein 2 and  **tau** .  Ref: 8186897 Virchows Arch, 1994
- Diffuse  **synaptophysin**  positivity was seen, accentuated along the borders of pleomorphic cells and in the rosettes, as well as diffuse interstitial and or cytoplasmic expression of neuron specific enolase, PGP 9.5 and  **tau** .  Ref: 7847074 Acta Neuropathol (Berl), 1994

**Parallel relationship** (e.g. studied together, co-existance, homology, etc.)

- ... reactivity for microtubule associated protein 2 MAP2,  **tau** , the 200 kilodalton isoform of neurofilament protein, neuron specific enolase and  **synaptophysin**  was primarily seen in maturing neurons.  Ref: 2586719 Neuropathol Appl NeurobiolNeuropathol Appl Neurobiol, 1994
- This investigation concerns the expression of paired helical filaments,  **tau**  protein, ubiquitin, beta amyloid protein, and  **synaptophysin**  in the hippocampus of patients with parkinsonism dementia complex on Guam PDC and Alzheimer s disease.  Ref: 1953965 J Geriatr Psychiatry NeurolJ Geriatr Psychiatry Neurol, 1994
- **Synaptophysin** , GAP 43, ubiquitin or  **tau**  did not label any normal or injured axons in this study.  Ref: 7847072 Acta Neuropathol (Berl), 1994
- were strongly reactive for neuron specific enolase,  **synaptophysin** , and neuronal cytoskeletal proteins 68 and 200 kd subunits of neurofilament protein, microtubule associated protein 2, and  **tau** .  Ref: 2757489 Arch Pathol Lab Med, 1989
- No positive reactions were obtained with antibodies against heat shock protein 27 HSP 27, HSP 72, actin, vimentin, desmin, cytokeratin, myelin basic protein, beta tubulin, MAP 2,  **tau**  protein, paired helical filament, phosphorylated neurofilament protein NFP, nonphosphorylated NFP,  **synaptophysin** , cathepsin D, alpha 1 antichymotrypsin, alpha 1 antitrypsin and basic fibroblast growth factor.  Ref: 1332366 Acta Neuropathol (Berl), 1992
- Examination of synaptic and cytoskeletal markers, i.e.,  **synaptophysin** , GAP 43, MAP 2,  **Tau**  1 and amyloid precursor protein, did not reveal any signs for neuronal reorganization or sprouting.  Ref: 7802968 J Chem Neuroanat, 1994
- This study also showed that microwave antigen retrieval significantly enhances the immunoreactivity of SNAP 25, chromogranin A,  **synaptophysin** , GAP 43, ubiquitin and  **tau** , in addition to that of beta APP, in formalin fixed, paraffin embedded tissue, and reveals NF68 antigenicity where it was not previously detectable.  Ref: 7847072 Acta Neuropathol (Berl), 1994
- Dystrophic neurites in the OE were GAP 43 positive, but they also expressed proteins that were not detected in normal olfactory nervesi.e.,  **synaptophysin** , MAP2,  **tau** , peripherin, NF proteins.  Ref: 1723988 J Comp Neurol, 1991
- Mature ganglion cells were also immunoreactive for proteins associated with the neuronal cytoskeleton including microtubule associated proteins, MAP2 and  **tau** , and higher molecular weight phosphorylated and non phosphorylated neurofilament subunits, neuron specific enolase, and  **synaptophysin** .  Ref: 2817080 Am J Pathol, 1989
- The markers studied include  **tau**  protein, ubiquitin, beta proteins,  **synaptophysin** , calcineurin, Met enkephalin, substance P and tyrosine hydroxylase.  Ref: 1583817 Keio J Med, 1992
- Following growth arrest, the cells display a neuron like morphology and express  **tau**  and  **synaptophysin** , two proteins synthesized in differentiating neurons.  Ref: 8475123 Proc Natl Acad Sci U S A, 1993
- Neurons were present, as determined by immunostaining with antibodies to 4 neuron specific proteins neuron specific enolase, microtubule associated protein 2,  **tau**  protein and  **synaptophysin** .  Ref: 2350878 Brain Res Dev Brain Res, 1990
- As in the AD hippocampus, the SPs detected by conventional stains in five of the 10 demented subjects contained the beta AP and flanking domains in beta APPs as well as epitopes in  **tau** , neurofilament proteins, and  **synaptophysin** .  Ref: 1377804 Neurology, 1992
